# Reviewer acknowledgement 2014

**DOI:** 10.1186/s13018-015-0167-y

**Published:** 2015-02-03

**Authors:** Anish Raj Kadakia, Nicola Maffulli

**Affiliations:** Northwestern University, Evanston, IL USA; Queen Mary University of London, London, UK

## Abstract

The Editors of *Journal of Orthopaedic Surgery and Research* would like to thank all our reviewers who have contributed to the journal in Volume 8 (2014).

**Michele Abate**

Italy

**Kashif Abbas**

Pakistan

**Ahmed Abdelazeem**

Egypt

**Huston Adkisson**

USA

**Syed Kamran Ahmed**

Hong Kong

**Yasushi Akamatsu**

Japan

**Tarek Aly**

Egypt

**Derek Amanatullah**

USA

**Chayanin Angthong**

Thailand

**Chandrasekaran Annamalai**

India

**Petros Antonarakos**

Greece

**Mitsuhiro Aoki**

Japan

**Theerachai Apivatthakakul**

Thailand

**Yasuto Araki**

Japan

**Chandra Aram**

USA

**Mateen Arastu**

United Kingdom

**Paul Arnold**

USA

**Apichat Asavamongkolkul**

Thailand

**Siamak Asgari**

Germany

**Bekir Atik**

Turkey

**Henry Dushan Edward Atkinson**

United Kingdom

**Marco Atteritano**

Italy

**Emmanuel Audenaert**

Belgium

**Ismael Auñon-Martin**

Spain

**Kyoko Baba**

Japan

**Goo Hyun Baek**

South Korea

**Vaibhav Bagaria**

India

**Clemens Baier**

Germany

**Peter Balcarek**

Germany

**Maurice Balke**

Germany

**Louisa Banks**

United Kingdom

**Timothy Barlow**

United Kingdom

**Greg Barritt**

Australia

**Anna Batistatou**

Greece

**Christoph Becher**

Germany

**Kimon Bekelis**

USA

**Mazllum Belegu**

Kosovo

**Claudio Belvedere**

Italy

**Anna C Berardi**

Italy

**Jean-Marie Berthelot**

France

**Yu Binsheng**

China

**James Binski**

USA

**Christof Birkenmaier**

Germany

**Jose Blanco**

United Kingdom

**Noam Bor**

Israel

**Lars Borris**

Denmark

**Mauro Bruzzone**

Argentina

**Martin Buttaro**

Argentina

**David Buxton**

USA

**Donita Bylski-Austrow**

USA

**Giorgio Maria Calori**

Italy

**Dominic Carreira**

USA

**Leah Carreon**

USA

**James Cashman**

Ireland

**Byron Chalidis**

Greece

**Jun-Dong Chang**

South Korea

**Shi-Min Chang**

China

**Nattapon Chantarapanich**

Thailand

**Charalambos Charalambous**

United Kingdom

**Vikram Chatrath**

USA

**Wen Ming Chen**

Australia

**Sally Cheng**

Hong Kong

**Louis Cheung**

Hong Kong

**Zachary Child**

USA

**Gun Choi**

South Korea

**Chantelot Chritstophe**

France

**Soo Yong Chua**

Singapore

**Bavornrit Chuckpaiwong**

Thailand

**Kook Jin Chung**

South Korea

**Moises Cohen**

Brazil

**Celine Colnot**

France

**Demitri Constantinou**

South Africa

**Fernando Corella**

Spain

**Aurélien Courvoisier**

France

**Raghuveer CV**

India

**Haleh Dadgostar**

Iran

**Adrien Daigeler**

Germany

**Mohammad Daneshi**

United Kingdom

**Michael Daubs**

USA

**Ronnie Davies**

United Kingdom

**Derik Davis**

USA

**Michael Dayton**

USA

**Masataka Deie**

Japan

**Hendrik Delport**

Belgium

**Carlos Dias**

Portugal

**Thomas Dienstknecht**

Germany

**Xavier Duralde**

USA

**Nathaniel Dyment**

USA

**Turgay Efe**

Germany

**Carl Ekholm**

Sweden

**Richard Ekstrom**

USA

**Bassel El-Osta**

United Kingdom

**Mehmet Erdil**

Turkey

**Mark Ereth**

USA

**Bengt I Eriksson**

Sweden

**Irfan Esenkaya**

Turkey

**Arezoo Eshraghi**

Malaysia

**Christina Esposito**

USA

**Jonatasce Esteves**

Brazil

**Emmanuel Estrella**

Philippines

**Filippo Familiari**

Italy

**Chi Ho Jason Fan**

Hong Kong

**Florian Fankhauser**

Austria

**Carlos Manuel Feced Olmos**

Spain

**Matheus Feitosa**

Brazil

**Justin Fernandez**

New Zealand

**Mariano Fernandez-Fairen**

Spain

**Giuseppe Filardo**

Italy

**Fernando Fonseca**

Portugal

**Jochen Franke**

Germany

**Simon Frostick**

United Kingdom

**MT Galloway**

USA

**Paolo Gargiulo**

Iceland

**Juerg Andreas Gasser**

Switzerland

**Barbara Gawronska-Kozak**

Poland

**Sandro Giannini**

Italy

**Jennifer Giuffre**

Canada

**Maciej Glowacki**

Poland

**Alejandro Gomez-Rice**

Spain

**Vinodkumar Gopaldasani**

Australia

**Sibylle Grad**

Switzerland

**Jove Graham**

USA

**Alberto Grassi**

Italy

**Francesco Grassi**

Italy

**Kirill Gromov**

Denmark

**Liam Grover**

United Kingdom

**Dong Yun Gu**

China

**Enpeng Gu**

China

**Andrew Hall**

United Kingdom

**Jaroslava Halper**

USA

**Didier Hannouche**

France

**Birgit Hanusch**

United Kingdom

**Furqan Haq**

USA

**Seyed Isaac Hashemy**

Iran

**Jun Hashimoto**

Japan

**Thomas Heyse**

Germany

**Pedro Hinarejos**

Spain

**James Hlavacek**

USA

**Florian Högel**

Germany

**Boris Michael Holzapfel**

Australia

**Ami Hommel**

Sweden

**Gary Hooper**

New Zealand

**Zhou Houxue**

China

**Yong-cheng Hu**

China

**Yingqi Hua**

China

**Jia Hua**

United Kingdom

**DaWei Huang**

Armenia

**Yuguang Huang**

China

**Leung-kim Hung**

Hong Kong

**Kousuke Iba**

Japan

**Satoshi Ikemura**

Japan

**Yuuki Imai**

Japan

**Gen Inoue**

Japan

**Cengiz Isik**

Turkey

**Yasuo Ito**

Japan

**Yoshiaki Itoigawa**

Japan

**Paul Ivancic**

USA

**Heidrun Jablonski**

Germany

**Michal Jagielski**

Germany

**Vinay Jasani**

United Kingdom

**Feng Ji**

China

**Liangjun Jiang**

China

**Nipun Jindal**

India

**Eleanor Jones**

United Kingdom

**I-Ming Jou**

Taiwan

**Sung Taek Jung**

South Korea

**Hiroshi Kaji**

Japan

**Satishchandra Kale**

India

**Umadevi Kandalam**

USA

**Tuncay Kaner**

Turkey

**Pengde Kang**

China

**Viktor Kanicky**

Czech Republic

**Eduardo Kanterewicz**

Spain

**Sinan Karaca**

Turkey

**Levent Karapinar**

Turkey

**Orestis Katsamenis**

United Kingdom

**Yoshiharu Kawaguchi**

Japan

**Michal Kedzierski**

Poland

**Emily Keener**

USA

**Byeongmo Kim**

USA

**Seung-Ho Kim**

South Korea

**Yoshimori Kiriyama**

Japan

**Thorsten Kirsch**

USA

**Hans-Michael Klinger**

Germany

**Peter Kloen**

The Netherlands

**Daisuke Koga**

Japan

**Ender Koktekir**

Turkey

**Alexander Kolb**

Austria

**Marina Komrakova**

Germany

**Rinco Koorevaar**

The Netherlands

**Kamil Cagri Kose**

Turkey

**Nusret Kose**

Turkey

**Karel Koudela**

Czech Republic

**David Kovacevic**

USA

**Ursula Krotscheck**

USA

**Sebastian Kuhn**

Germany

**Jean-Louis Labbe**

New Caledonia

**Kuo-An Lai**

Taiwan

**Po-Liang Lai**

Taiwan

**Alessandro Landi**

Italy

**Billy Kan-Yip Law**

Hong Kong

**Chianher Lee**

Taiwan

**Peter Lee**

Australia

**Kirsten Legerlotz**

Ukraine

**Mario Lenza**

Brazil

**Aleksndar Lesic**

Serbia

**Zhi-jun Li**

China

**Hui Li**

China

**Jinqian Liang**

China

**Chia-Ying Lin**

USA

**Senshang Lin**

USA

**James Linklater**

Australia

**Lukas Lisowski**

The Netherlands

**Hwa-Chang Liu**

Taiwan

**Jun Liu**

China

**Heinz Lohrer**

Germany

**Giovanni Lovisetti**

Italy

**Guntur Luis**

Malaysia

**Hsiao-Li Ma**

Taiwan

**Richard Ma**

USA

**Xinlong Ma**

China

**Tomislav Madarevic**

Croatia

**Aditya Maheshwari**

USA

**Jean-Yves Maigne**

France

**Mark Manoso**

USA

**Zhi Mao**

China

**Rex Marco**

USA

**Ingo Marzi**

Germany

**Baratali Mashkani**

Iran

**Shuichi Matsuda**

Japan

**Ville Mattila**

Finland

**Uwe Max Mauer**

Germany

**Andreas Mavrogenis**

Greece

**Susanne Mayer-Wagner**

Germany

**Jeffrey McLaughlin**

USA

**Margaret McQueen**

United Kingdom

**Marrigje Meijer**

The Netherlands

**Hans Jörg Meisel**

Germany

**Duncan Meuffels**

The Netherlands

**Thomas Mittlmeier**

Germany

**Hideki Mizu-Uchi**

Japan

**Manuel Monteagudo**

Spain

**Silas Motsitsi**

South Africa

**Scott Mubarak**

USA

**Dayal Mukherjee**

United Kingdom

**Matthias Münzberg**

Germany

**Urs Munzinger**

Switzerland

**Arata Nakajima**

Japan

**Rahul Nath**

USA

**Eric Nauman**

USA

**Nuno Neves**

Portugal

**Toshihiko Nishisho**

Japan

**Angela Notarnicola**

Italy

**Alan Nurden**

France

**Richard Walter Nyffeler**

Switzerland

**Kensuke Ochi**

Japan

**Onder Ofluoglu**

Turkey

**John O'Hara**

United Kingdom

**Hirotsugu Ohashi**

Japan

**Atsushi Okawa**

Japan

**Francesco Oliva**

Italy

**Rene Olivares-Navarrete**

USA

**Bo Sanderhoff Olsen**

Denmark

**Farzad Omidi-Kashani**

Iran

**Georg Omlor**

Germany

**Alvin Ong**

USA

**Nada Orsolic**

Croatia

**Mohamed Osman**

Egypt

**Georg Osterhoff**

Switzerland

**Hong Wei Ouyang**

China

**Türker Özkan**

Turkey

**Johnny Padulo**

Italy

**Jeya Palan**

United Kingdom

**Alessio Palumbo**

Italy

**Peter Panfilov**

Russia

**Stamatios Papadakis**

Greece

**Rocco Papalia**

Italy

**Daniel Park**

USA

**Moon Seok Park**

South Korea

**David Parker**

Australia

**Martyn Parker**

United Kingdom

**Javad Parvizi**

USA

**Fuxing Pei**

China

**Hao Peng**

China

**Jiang Peng**

China

**Rodrigo Pesantez**

Colombia

**Ákos Pethes**

Hungary

**Stefano Petrillo**

Italy

**Eleonora Piccirilli**

Italy

**Oliver Pieske**

Germany

**Matthias Pietschmann**

Germany

**Kevin Plancher**

USA

**Jashvant Poeran**

USA

**Sina Pourtaheri**

USA

**Rabindra Pradhan**

Nepal

**Guixing Qiu**

China

**Vafa Rahimi-Movaghar**

Iran

**Mohammad Razi**

Iran

**Felipe Reis**

Brazil

**Giuseppina Resmini**

Italy

**Michael Ries**

USA

**Matthew Robinson**

USA

**Yohan Robinson**

Sweden

**Pol Maria Rommens**

Germany

**Alfredo Ronca**

Italy

**Steffen Bernd Rosslenbroich**

Germany

**Brice Rubens Duval**

France

**Hannes Rudiger**

Switzerland

**Pietro Ruggieri**

Italy

**Jurgen Runge**

The Netherlands

**Luigi Sabatini**

Italy

**Manuel Sabeti**

Austria

**Tomoyuki Saito**

Japan

**Milan Samardjiski**

Macedonia

**Parag Sancheti**

India

**Hiroshi Sasaki**

Japan

**Terence Savaridas**

United Kingdom

**Olga Savvidou**

Greece

**Mario Francesco Carlo Scaglioni**

Italy

**T Schepers**

The Netherlands

**Max Joseph Scheyerer**

Switzerland

**Thomas Schildhauer**

Germany

**Aaron Schindeler**

Australia

**Corey Scholes**

Australia

**Lew Schon**

USA

**Verena Schreiber**

USA

**Leah Schulte**

USA

**Gundula Schulze-Tanzil**

Germany

**Ran Schwarzkopf**

USA

**Manuel Segura-Trepichio**

Spain

**Jonathan Sembrano**

USA

**Peng Shang**

China

**Sadhna Sharma**

India

**Wun-Jer Shen**

Taiwan

**Dufang Shi**

China

**Won Yong Shon**

South Korea

**Sandeep Shrivastava**

India

**Jerzy Siepak**

Poland

**Koopong Siribumrungwong**

Thailand

**Sarah Snelling**

United Kingdom

**Reuben Soh**

Singapore

**Henrik Sönnergren**

Sweden

**Philip Stahel**

USA

**Sarah Stapley**

United Kingdom

**Hans Werner Stedtfeld**

Germany

**Arnd Steinbrueck**

Germany

**Karl Stoffel**

Switzerland

**Hiroyuki Sugaya**

Japan

**Abdul Razak Sulaiman**

Malaysia

**Dong Sun**

China

**Nobuyuki Suzuki**

Japan

**Yoshiaki Tabuchi**

Japan

**Norimasa Takahashi**

Japan

**Sunao Takeshita**

Japan

**Boonsin Tangtrakulwanich**

Thailand

**Umberto Tarantino**

Italy

**Sarunas Tarasevicius**

Lithuania

**Francisco José Tarazona Santabalbina**

Spain

**Yoshinori Terashima**

Japan

**Stefan Thomas**

Switzerland

**Marco Tinius**

Germany

**Thomas Tischer**

Germany

**Serdar Toker**

Turkey

**Carlos Torrens**

Spain

**Ismail Remzi Tozun**

Turkey

**Andrea Trescot**

USA

**Sujit Tripathy**

India

**Tung-Hu Tsai**

Taiwan

**Chongqi Tu**

China

**Ibrahim Tuncay**

Turkey

**Yuji Uchio**

Japan

**Akin Ugras**

Turkey

**Ken Urabe**

Japan

**James Urbaniak**

USA

**Mohammad Urrazaq**

Pakistan

**Ger Van Olden**

The Netherlands

**Dirkjan Veeger**

The Netherlands

**Steven Velkes**

Israel

**Petri Virolainen**

Finland

**Emilio Wagner**

Chile

**Ola Wahlstrom**

Sweden

**Norimitsu Wakao**

Japan

**Hai-Qiang Wang**

China

**Jeffrey Wang**

USA

**Qing Wang**

China

**Lin Wang**

China

**Jun-Wen Wang**

Taiwan

**Yisheng Wang**

China

**Xiupeng Wang**

Japan

**He Wangjiao**

China

**James Ward**

USA

**Russell Warren**

USA

**Tim Weber**

Germany

**Zhang Wei**

China

**Tina Stromdal Wik**

Norway

**Robert Wilkins**

United Kingdom

**Simon Wimsey**

United Kingdom

**Philip Wolinsky**

USA

**Chi-Chuan Wu**

Taiwan

**Dan Xing**

China

**Hui-feng Yang**

China

**Yunfeng Yao**

China

**Muneyoshi Yasuda**

Japan

**Takao Yasuhara**

Japan

**Hamidreza Yazdi**

Iran

**Hong Yi**

China

**Guangfu Yin**

China

**Go Yoshida**

Japan

**Xiu-Chun Yu**

China

**Mohammad Javad Zehtab**

Iran

**Johannes Zellner**

Germany

**Tao Zhang**

China

**Ying-ze Zhang**

China

**Chunfeng Zhao**

USA

**Sun Zhenhui**

China

**Bing Zhou**

China

**Wierd Zijlstra**

The Netherlands

**Michael G Zywiel**

Canada

